# Pediatric onset neuronal ceroid lipofuscinoses: Unraveling clinical and genetic specifications

**DOI:** 10.12669/pjms.40.8.8006

**Published:** 2024-09

**Authors:** Saher Gul Ahdi, Javeria Raza Alvi, Azeem Ashfaq, Tipu Sultan

**Affiliations:** 1 Dr. Saher Gul Ahdi University of Child Health Sciences, The Children’s Hospital, Lahore, Pakistan; 2 Dr. Javeria Raza Alvi University of Child Health Sciences, The Children’s Hospital, Lahore, Pakistan; 3 Dr. Azeem Ashfaq University of Child Health Sciences, The Children’s Hospital, Lahore, Pakistan; 4 Dr. Tipu Sultan University of Child Health Sciences, The Children’s Hospital, Lahore, Pakistan

**Keywords:** Genotype, Myoclonic Jerks, Neuronal ceroid lipofuscinoses, Optic Atrophy, Phenotype

## Abstract

**Objective:**

To unravel the clinical and genetic specifications of Neuronal ceroid lipofuscinosis (NCL).

**Methods:**

This is a retrospective cross-sectional study conducted in the Department of Pediatric Neurology Children Hospital and University of Child Health Sciences, Lahore, Pakistan from March 2017 to March 2022. The primary outcome was to measure genotype-phenotype correlation by segregation of phenotypes according to genotype. The secondary outcomes included a correlation between genotype and distribution of age(s) of onset.

**Results:**

One hundred fifty three patients clinically diagnosed with NCL underwent genetic testing and pathologic mutation was identified in 32.7% of patients. About 59.6% were male and 37.2% had an affected sibling. The median age was 5.46±1.95 years at the onset of the first symptom i.e., myoclonic seizures in 68%, and motor difficulty in 24%. Other features found were global developmental delay (56%), hypotonia (23%), visual impairment (80%), ataxia (22%), and disc pallor (56%). The most common type was CLN6 (Ceroid lipofuscinosis neuronal) (42%), CLN2 (16%) followed by CLN7 (12%). When 50 patients with recognized mutations were compared with 103 patients with no mutation, family history (p=0.049), early visual loss (p=0.016), hypotonia (p=0.001), white matter signals (p=0.026) and pan-atrophy(p=0.047) was statistically significant in the genetically confirmed NCL. Multiple pairwise comparisons indicated that the estimated age of onset for the CLN1 and CLN2 mutation group was significantly lower than other genotypes including CLN6 (p 0.012), CLN10 (p 0.007) and CLN12 (p 0.007).

**Conclusion:**

Following a detailed review of NCL symptomatology, a clinically-oriented approach should be used for a rapid diagnosis with confirmation by targeted molecular testing for future genetic counseling.

## INTRODUCTION

Neuronal ceroid lipofuscinoses (NCL) is a group of rare inherited lysosomal storage disorders leading to fatal progressive neurodegeneration. It is characterized by abnormal accumulation of auto-fluorescent material in lysosomes of the cells affecting mainly the retina and gray matter of the cerebral cortex.^1^ It has been recognized as one of the most frequent childhood-onset neurodegenerative pathologies with an incidence of 1.6–2.4/100 000 in America^2^ involving all ages and either gender with a global distribution.^3^

Presenting clinical features are a combination of epilepsy, psychomotor regression on previously age-appropriate milestones, cognitive decline and loss of vision.^4^ NCL is inherited in an autosomal recessive pattern showing a large clinical and genetic heterogeneity.^5^ To date, more than 500 genetic variants in 13 different forms have been identified. These are classified according to the age of onset into the congenital, infantile, late infantile, juvenile or adult form, and/or the affected gene.^6^ These genes encode lysosomal enzymes (PPT1/CLN1, TPP1/CLN2, CTSD/CLN10, CTSF/CLN13), protein in the secretory pathway (GRN/CLN11), two cytoplasmic proteins (DNAJC5/CLN4 and KCTD7/ CLN14), a soluble lysosomal protein (CLN5) and many transmembrane proteins with different subcellular locations (CLN3, CLN6, MFSD8/CLN7, CLN8 and ATP13A2/CLN12). The gene responsible for CLN9 has not been identified.^2^ Exact mechanism of how deficiencies of proteins lead to the accumulation of lysosomal storage material and subsequent neuro-degeneration has not yet been understood.^7^

This information is lacking from Pakistan wherein the arrival of whole-exome sequencing has modified diagnostics although challenges remain concerning expenses and the absence of local population-based data. Through this study we want to unravel the clinical and genetic specifications associated with different types of Neuronal ceroid lipofuscinosis utilizing advanced genetic investigation i.e., Whole Exome Sequencing (WES).

## METHODS

It was a hospital based retrospective descriptive study conducted in the Department of Pediatric Neurology at the Children’s Hospital and University of Child Health Sciences, Lahore, Pakistan from March 2017 to March 2022. Sampling technique was non-probability consecutive

### Ethical Approval:

It was obtained from the institutional review board (2022-595-CH-UCHS), dated September 16, 2022.

### Inclusion & Exclusion Criteria:

All the confirmed patients of NCL via genetic testing during the selected study period were included. Patients with psychomotor regression, myoclonic jerks and visual loss due to causes other than NCL were excluded.

For genetic testing, blood samples were taken from the probands after informed consent. DNA was extracted at UCL Queen Square Institute of Neurology (UK). All the data was obtained using a predesigned proforma. Demographic details and clinical variables of interest including age at onset, gender, initial clinical feature(s) and its evolution, associated features and genetic mutations were extracted for individual patients. Moreover, the type of epilepsy as per the International League Against Epilepsy classification, developmental age and magnetic resonance imaging (MRI) results were also evaluated. The primary outcome measure was genotype-phenotype correlation as measured by segregation of clinical phenotypes according to genotype. The secondary outcomes included a correlation between genotype and distribution of age(s) of onset.

### Statistical analysis:

Data was interpreted using SPSS version 25. The relationship between genotypes (CLN1, CLN2, CLN3, CLN5, CLN6, CLN7, CLN8, CLN10 and CLN12) and phenotypes (infantile, late infantile and juvenile) was examined using chi-square test. One-way analysis of variance was conducted with age of onset as the dependent variable and genotype being the explanatory variable. To resist the effect of possible outliers, a robust analysis of variance was used. Pair-wise differences between the genotypes were computed, and to account for multiple testing, a false discovery rate was used with a family-wise error rate set at 0.05.

## RESULTS

A total of 153 patients of NCL were identified on clinical grounds who underwent genetic testing and a pathologic mutation was identified in 32.7% (n=50) of patients. Male gender was predominant in 59.6% (n=93) and 37.2% (n=58) patients had an affected sibling. The median age was 5.46 ± 1.95 years at the onset of the first clinical symptom. The most common initial symptoms were seizures in 68% (n=34), motor difficulty in 24% (n=12) followed by cognitive decline and language difficulty in 8% (n=4). The median age of death was found to be 10.71 ± 2.54 years and the time lapse between the onset of the first symptom and time of death was calculated to be 5.28 ± 1.69 years. Salient clinical features and neuroimaging findings in clinically vs genetically confirmed NCL are explained in Table-I and neuroimaging in regard to NCL diagnosis made clinically vs genetically.

**Table-I:** Comparison of clinical features and investigation of NCL patients diagnosed on genetic vs clinical grounds. NCL-Neuronal ceroid lipofuscinosis; GDD- Global developmental delay; GTC- Generalized tonic-clonic.

Clinical Features, and investigations	Total patients N=153(%)	Number of patients with molecular genetic diagnosis of NCL n=50(%)	Number of patients with no molecular genetic diagnosis of NCL n=103(%)	p-Value
***Salient neurological features at outset***				
GDD	75 (49.0%)	28 (56.0%)	47 (45.6%)	0.139
Hypotonia	78 (50.9%)	35 (22.8%)	43 (41.7%)	0.001
Early visual impairment	83 (54.2%)	42 (80.7%)	41 (39.8%)	0.0001
Ataxia	37(24%)	11(22%)	26(55.5%)	0.083
Tremors	76(49.6%)	22(44%)	54(52.4%)	0.198
***Ophthalmological examination***				
Retinitis Pigmentosa	7(4.5%)	5(10%)	2 (1.9%)	
Pale disc	64(41.8%)	28(56%)	36(34.9%)	
***Seizure semiology at outset***				
GTC	75 (49.0%)	27 (54.0%)	48 (46.6%)	0.230
Myoclonic	81 (52.9%)	33 (66.0%)	42 (40.7%)	0.016
Absence	-	-	-	-
Infantile spasms	-	-	-	-
***Neuroimaging***				
Cerebral and cerebellar atrophy	79 (51.6%)	31 (62.0%)	48 (46.6%)	0.047
T2W hyperintensities	61 (39.8%)	24 (48.0%)	37 (35.9%)	0.026
Corpus Callosum abnormalities	28 (18.3%)	7 (14.0%)	21 (20.3%)	0.981

### Classification of NCL:

**Fig.1:**
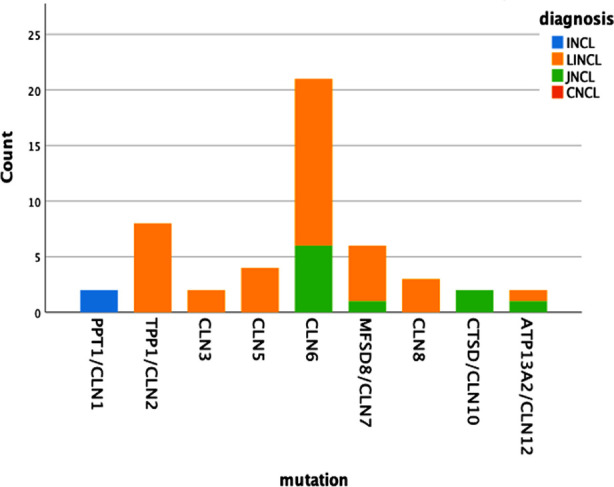
The distribution of reported clinical phenotypes by genotype.

Regarding the classification, Fig.1 gives an outlook of the common gene variants and age of presentation. It was observed that two patients with infantile onset NCL were reported to have mutation in PPT1/CLN1. Therefore, the analysis for the association between genotypes and phenotype was limited to late infantile and juvenile phenotypes. A significant difference in the clustering of genotypes was observed according to the clinical phenotype (P < 0.0001) Table-I. CLN2, CLN3, CLN5 and CLN8 genotypes were significantly more likely to present as infantile NCL (including late infantile) while CLN10 was the only genotype more likely to present as juvenile NCL. Other mutations like CLN6, MFSD/CLN7, and ATP13A2/CLN12 indicated a bimodal presentation with both late infantile and juvenile onset NCL.

**Fig.2:**
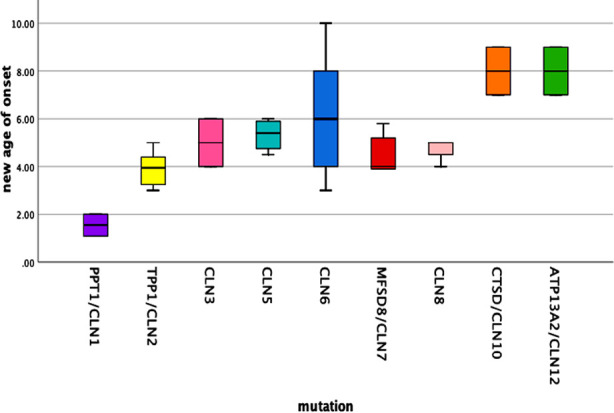
The age of onset in patients with neuronal ceroid lipofuscinoses by genotype.

### Genotype and Phenotype:

When 50 patients with recognized mutations were compared with 103 patients who were not found to carry any mutation, family history with an affected sibling (p= 0.049), early visual loss (p=0.016) and hypotonia (p=0.001) was statistically significant in the genetically confirmed NCL (Table-I). Moreover, the most common seizure type was myoclonic jerks significantly found in genetically confirmed NCL. On neuroimaging pan-atrophy (p=0.047) and T2W peritrigonal and periventricular hyperintensities (p=0.026) were prominent findings associated with positive genetic mutation.

### Genotype and age of onset:

Pairwise comparisons were performed to analyze the association between age of onset and different genotypes using robust methods as previously discussed. The estimated age of onset as a function of genotype is explained in Table-II (a-b). Multiple pairwise comparisons indicated that the estimated age (years) of onset for the CLN1 mutation group (1.55 ± 0.460) was significantly lower than other genotypes including CLN6 (6.08 ± 0.349, p 0.012), CLN10 (8.00 ± 1.00, p 0.007) and CLN12 (4.54 ± 1.00, p 0.007). Likewise, the estimated age of onset for CLN2 mutation group (3.90 ± 0.261) was also significantly lower than CLN6 (6.08 ± 0.349, p 0.049), CLN10 (8.00 ± 1.00, p 0.055) and CLN12 (4.54 ± 1.00, p 0.055). Although the age of onset for the CLN1 genotype was also lower than the CLN2 (3.90 ± 0.261), CLN3 (5.00 ± 1.00), CLN5 (5.32 ± 0.69), CLN7 (4.47± 0.33) and CLN8 (4.66 ± 0.33) genotypes, the difference did not attain statistical significance (p 0.649, 0.456, 0.174, 0.408 and 0.470 respectively).

**Table-II(a):** The Correlation Between Age of Onset and Genotype in Patients with Neuronal Ceroid Lipofuscinoses and Genotype.

Gene	Mean age of onset	Standard error	95% confidence interval for mean	

			Lower bound	Upper bound
PPT1/CLN1	1.55	0.460	-4.29	7.394
TPP1/CLN2	3.90	0.261	3.282	4.517
CLN3	5.00	1.000	-7.706	17.706
CLN5	5.32	0.349	4.212	6.437
CLN6	6.08	0.462	5.120	7.050
MFSD8/CLN7	4.47	0.335	3.607	5.332
CLN8	4.66	0.333	3.232	6.100
CTSD/CLN10	8.00	1.000	-4.706	20.706
ATP13A2/CLN12	8.00	1.000	-4.706	20.706

**Table-II(b):** The Correlation Between type of NCL with Genotype.

	CLN2	CLN3	CLN5	CLN6	CLN7	CLN8	CLN10	CLN12
CLN1	0.649	0.456	0.174	0.012*	0.408	0.470	0.007*	0.007*
CLN2	-	0.994	0.871	0.049*	0.999	0.998	0.050*	0.050*
CLN3	-	-	1.000	0.991	1.000	1.000	0.638	0.638
CLN5	-	-	-	0.993	0.995	1.000	0.602	0.602
CLN6	-	-	-	-	0.440	0.879	0.793	0.793
CLN7	-	-	-	-	-	1.000	0.182	0.182
CLN8	-	-	-	-	-	-	0.380	0.380
CLN10	-	-	-	-	-	-	-	1.000

## DISCUSSION

We retrospectively reviewed genetically confirmed 50 children with Neuronal ceroid lipofuscinoses out of 153 suspected NCL patients on clinical grounds (psychomotor regression, progressive visual impairment, myoclonic jerks and cerebral and cerebellar atrophy on neuroimaging). We found the median age of onset to be 5.46 ± 1.95 years in our study population which correlates with the median age of onset documented by Sinha S et al. i.e., 5.9 ± 9.1 years.^8^ Males were most commonly affected in our cohort which has been consistently found in the previous studies as well.^9^,^10^ The degree of consanguinity with affected siblings had more diagnostic yield on direct sanger sequencing in CLN genes, also evident in our cohort showing 37.2% of patients with an affected sibling.^11^,^12^ Regarding the disease classification, there is a wide variability in the presentation of different CLN gene variants possibly due to distinctive ethnicity worldwide. Although CLN3 is considered to be the most common type of NCL in different parts of the world^13^, we found CLN6 to be the most common type in our cohort having a bimodal onset (late infantile and juvenile) followed by CLN2, contrary to the literature. CLN2, apart from CLN7, was also found to be a common type in the Russian population.^14^ CLN1 usually presents under two years of age, with some studies showing late-onset variants and our two patients of CLN1 also presented in early infancy.^9^ All the patients with mutations in genes including TPP1/CLN2, CLN3, CLN5 and CLN8 presented with a late infantile NCL phenotype in our study population.^15^ Most of them presented with speech delay, seizures and motor decline along with progressive loss of vision. Four patients presented with progressive learning and language difficulty, motor difficulty and seizures ending in complete visual loss. Guerreiro et al., documented CLN6 mutation presenting as late infantile form in two Pakistani families.^16^ CLN2, CLN5 and CLN8 gene mutations presented as late infantile but CLN3 presented as a juvenile-onset disease characterized by rapid progressive loss of vision as the first presenting symptom.^13^,^14^ Also Sher M et al., documented CLN3 presenting as a juvenile form in large Pakistani families. 17 Apart from CLN6, MFSD8/CLN7, ATP13A2/CLN12 also had a bimodal presentation, presenting both as late infantile and juvenile type of NCL in our study population.

We studied the clinical presentation only at the outset to identify how early and with what symptoms the different types of NCL can present. We found global developmental delay in 54% to start with followed by psychomotor regression in all the patients. Visual decline (80%) was the most common finding in our cohort consistent with previous literature.^12^,^13^ Although visual impairment is a very striking finding in NCL and Gowda et al found equivalent cases of retinal pigmentary (RP) changes, disc pallor and atrophy along with rare cases of bull’s eye maculopathy on detailed ophthalmological examination^9^, on the contrary, we had a significantly large number of cases with disc pallor (56%) while only a few cases with RP (10%). Jilani A et al, documented generalized tonic-clonic (p<0.0015), myoclonic (p<0.008) and partial (p<0.018) type of seizure activity as a common occurrence in NCL and was found significant in patients with genetically diagnosed NCL.^12^ In our study population, we found only myoclonic jerks (p<0.016) as a significant seizure type in genetically proven NCL (15%). Ebrahimi et al studied movement disorders associated with different types of NCL and found ataxia and resting tremors to be the most common ones specifically in CLN6, CLN2 and CLN14, similar to our set of patients.^18^ Moreover, on clinical examination findings Jilani A et al., found hypotonia (p<0.0017) and early visual impairment (p <0.0001) to be markedly significant in patients with a molecular genetic diagnosis of NCL.^12^ We also encountered hypotonia as a significant feature (p<0.001) along with early visual loss (p<0.0001) in patients of genetically proven NCL as compared to the patients with no molecular diagnosis.

Neuroimaging spectrum is wide starting from normal MRI if done at early ages, followed by marked cerebral and cerebellar atrophy in almost all cases as studied by Gowda et al.^9^ Apart from these, Biswas et al studied the ever-expanding neuroradiological phenotype over the decades with identification of white matter changes and hypointense T2W signal intensity in thalami as a sign of lysosomal storage disorder, also studied by Autti et al.^19^-^21^ We also found global atrophy (62%) followed by white matter changes (48%) similar to above-mentioned studies but also found corpus callosum abnormalities in 14% of our patients seldom described in the literature.

Neuronal Ceroid Lipofuscinoses is one of the most common neuro-degenerative brain having variable clinical phenotype and genetic heterogeneity rendering the diagnosis difficult.^15^,^22^ NCL has been well known to be presented with psychomotor regression, myoclonic seizures, visual impairment and a common endpoint of a vegetative state with cerebral and cerebellar atrophy.^6^ This is the largest study from our part of the world to the best of our knowledge that has unwavered different types of CLN gene variants according to our ethnicity and demography. Additionally, and differently, our large number of patients presented with developmental delay rather than regression at the outset, myoclonic jerks as the leading seizure type, strikingly more disc pallor than retinitis pigmentosa, and callosal abnormalities on MRI brain.

### Limitations:

It is a single centered study. It would have been more effective and applicable if a multicentered study was conducted. Also, that as being done for the first time for the Pakistani population on a large scale, no locally published data was available for the comparison of results.

## CONCLUSION

This study supports a distinct segregation of genotypes and clinical phenotypes within the NCL. Following a detailed historical review of the evolution of NCL symptomatology, a clinically-oriented approach should be used in describing how early symptoms and signs including seizures, psychomotor regression and visual impairment can appear. This may lead clinicians to a rapid clinical diagnosis with confirmation by targeted molecular testing avoiding the long diagnostic odyssey commonly observed and help in genetic counseling.

### Recommendations:

Thorough review of symptomatology including myoclonic seizures, psychomotor delay/regression and visual impairment with disc pallor can give early clues towards the diagnosis of NCL and thus, short of genetics, a clinically-oriented approach should be used.

### Authors` Contribution:

**SGA & JRA:** Equally contributed to the conception, design, data collection, interpretation, manuscript writing, editing and statistical analysis**.**

**JRA**: Responsible for integrity of research.

**AA & TS**: Manuscript editing.

**TS**: Review and final approval of manuscript.
